# Response to: Comment on “A Sustained Reduction in Serum Cholinesterase Enzyme Activity Predicts Patient Outcome following Sepsis”

**DOI:** 10.1155/2019/9258509

**Published:** 2019-08-26

**Authors:** Aleksandar R. Zivkovic, Thorsten Brenner

**Affiliations:** Department of Anesthesiology, Heidelberg University Hospital, Heidelberg, Germany

We thank Dr. Chiarla and Dr. Giovannini for their interest in our study regarding the role of reduced serum cholinesterase activity in predicting patient outcome following systemic inflammation and sepsis [1]. We agree with the raised points, and we acknowledge the need to investigate these questions further.

Drs. Chiarla and Giovannini commented on two aspects. The first one is a concern that the precise BChE activity levels which we stated in our manuscript [2] to be predictive of a higher risk for poor outcome in sepsis patients might not transfer precisely to other hospitals due to the diversity in commercially available test kits used across diagnostic laboratories. We agree with Dr. Chiarla and Dr. Giovannini. To offset this variability, the protocol used in our study implemented a point-of-care testing device allowing faster and straightforward bedside measurement of serum cholinesterase activity. As mentioned by Drs. Chiarla and Giovannini, reporting a precise cutoff value of BChE activity predictive of high risk is an essential step in the statistical analysis and scientific reporting of the obtained data [3]. We feel that the reported cutoff value might provide a basis for prospective data analysis, although interpreting BChE activity levels from diverse laboratory protocols remains a challenge. Thus, to overcome the problem of “translating” various reference ranges, we suggest using normalized activity values [2, 4, 5] similar to the 25% of the minimum normal range used by Drs. Chiarla and Giovannini.

The second point raised by Drs. Chiarla and Giovannini is that changes in BChE activity occur independent of illness severity following pleural and ascitic fluid loss or bleeding, which decreases BChE activity, or transfusion of fresh frozen plasma, which increases BChE activity levels. We agree with Drs. Chiarla and Giovannini that this is an important and often ignored fact that needs to be systematically considered in more detail [6, 7]. Paracentesis and thoracentesis, as well as fresh frozen plasma transfusion, should be included when analysing the course and dynamics of the serum cholinesterase activity levels in all patients with critical illness.

In conclusion, we appreciate the authors' interest in our study and acknowledge the raised questions as highly relevant. Furthermore, we endorse the use of the suggested regression formula for interpreting the activity change of serum cholinesterase in critical illness. Finally, we agree with Dr. Chiarla and Dr. Giovannini that serum cholinesterase activity change might play a paramount role in early risk stratification of patients with systemic inflammation and sepsis.

## References

[1] Chiarla C., Giovannini I. (2019). Comment on “A sustained reduction in serum cholinesterase enzyme activity predicts patient outcome following sepsis”. *Mediators of Inflammation*.

[2] Zivkovic A. R., Decker S. O., Zirnstein A. C. (2018). A sustained reduction in serum cholinesterase enzyme activity predicts patient outcome following sepsis. *Mediators of Inflammation*.

[3] Cummings P., Rivara F. P. (2003). Reporting statistical information in medical journal articles. *Archives of Pediatrics & Adolescent Medicine*.

[4] Zivkovic A. R., Bender J., Brenner T., Hofer S., Schmidt K. (2016). Reduced butyrylcholinesterase activity is an early indicator of trauma-induced acute systemic inflammatory response. *Journal of Inflammation Research*.

[5] Zivkovic A. R., Tourelle K. M., Brenner T., Weigand M. A., Hofer S., Schmidt K. (2017). Reduced serum cholinesterase activity indicates splenic modulation of the sterile inflammation. *Journal of Surgical Research*.

[6] Chiarla C., Giovannini I., Giuliante F., Vellone M., Ardito F., Nuzzo G. (2011). Plasma cholinesterase correlations in acute surgical and critical illness. *Minerva Chirurgica*.

[7] Garcia-Pachon E., Padilla-Navas I., Sanchez J. F., Jimenez B., Custardoy J. (1996). Pleural fluid to serum cholinesterase ratio for the separation of transudates and exudates. *Chest*.

